# Porosity of a Fast-Setting Mortar with Crystallization Admixture and Effect of a SA-PA Modification

**DOI:** 10.3390/ma15041542

**Published:** 2022-02-18

**Authors:** Oscar Cotini, Rosa Di Maggio, Daniel Tonelli, Roger Nascimben, Narges Ataollahi

**Affiliations:** Department of Civil, Environmental and Mechanical Engineering, University of Trento, 38123 Trento, Italy; rosa.dimaggio@unitn.it (R.D.M.); daniel.tonelli@unitn.it (D.T.); roger.nascimben@alumni.unitn.it (R.N.); narges.ataollahi@unitn.it (N.A.)

**Keywords:** porosity, superabsorbent polyacrylates, air permeability, water permeability, SEM imaging, mercury intrusion porosimetry

## Abstract

Air permeability measurements according to the Hagen–Poiseuille equation, Scanning Electron Microscopy (SEM) and mercury intrusion porosimetry (MIP) tests were conducted on samples of cementitious mortar at different curing times to study the correlation between the increased crystallization and their microstructure. The mortar samples were prepared with a commercial fast-setting premix containing calcium silicates and quartz. The average permeability coefficient (K) was 2.96 × 10^−15^ m^2^ after 3 days and decreased to 3.07 × 10^−17^ m^2^ after about one month. The continuous C-S-H nucleation in the capillary pores of the cement mortar changes their shape and improves the mortar’s impermeability. The SEM images showed the development of crystals that refine the pore size distribution of the cement paste, with more of the smallest pores, and fewer of the largest, as demonstrated by the MIP measurements. Adding a superabsorbent polyacrylate (SA-PA) in the amount of 0.5% wt of dry powder, without adding any extra water, makes a mortar less fluid but not faster-setting. Twenty-four hours after mixing and casting, it is still plastic and, with time, the pore size distribution differs from that of standard mortar. Over time in air, permeability remains high, but in water it could be low due to swelling of SA-PA residues.

## 1. Introduction

There is always a growing demand for impermeable concrete for building hydraulic structures, such as channels and dams, and for water containment generally [1,2]: the more impermeable the concrete, the more durable the structure. Although it may not be enough to prevent degradation in chemically aggressive environments [3,4,5,6,7], using impermeable concrete can reduce the degradation rate, and the associated maintenance costs and energy consumption. According to the norms and standards, a concrete structure is designed taking into consideration its nominal service life (which depends on the type of structure and environmental factors) and specifying an appropriate level of concrete permeability [8]. Current European standards (Eurocode 2 and EN 206:2016) require that designers indicate a concrete’s resistance to the penetration of water, as the most direct measure of its capacity to withstand the passage of fluids, harmful chemicals, and trivially carbon dioxide and oxygen, which cause deterioration in the cementitious matrix and steel reinforcement, respectively [9]. Resistance to water penetration can be measured on concrete prototypes, but hardly on the concrete in place.

There are at least two different transport mechanisms in concrete: diffusion and permeation. Diffusion occurs when there is a concentration gradient between the two faces of a porous material. Permeation occurs when a pressure gradient is created within the material [10]. In the latter case, it is not only the volume, but also the shape of the porosities that are important, and they are interdependent. All transport phenomena are correlated to the material’s porosity, which can be classified according to IUPAC recommendations: macro (d = pore diameter > 50 nm), meso (2 < d < 50 nm), or micro (d < 2 nm), though this last could more precisely be described as a nano level [11,12]. Porosity in concrete varies from macro-bubbles of air to capillary porosity and gel porosity. In terms of permeability, or the motion of air under hydrostatic pressure, and of transport phenomena in concrete in general, only meso- and macroporosity are important [13]. The permeability of concrete depends mainly on its internal connection state [14], which changes while hydration reactions and crystallization are underway. According to the Rose study, the speed and depth of water penetration in concrete materials depend on their microstructure and pore size [15]. Studying concrete permeability is therefore hugely significant because it is closely connected to the penetration of water, which reduces the pH value of the pore fluid and carries harmful ions, resulting in degradation and loss of mechanical strength. That said, these properties are hardly ever measured in concrete in place.

A common method for measuring concrete permeability to gases and fluids is to use a permeability cell to expose one side of a specimen to a fluid under pressure and measure the flow at the inlet or outlet [16]. The Italian standard UNI 1116400:2005 [17] describes the procedure for measuring the air permeability of concrete specimens under a pressure gradient in a steady state condition. To measure a concrete’s resistance to the penetration of aggressive agents, the maximum penetration depth of water under pressure inside hardened concrete could be measured according to EN 12390-8: 2019 [18], ascertaining its coefficient of permeability (K) using the Hagen–Poiseuille equation and its capillary absorption according to EN 13057:2002 [19].

Although air permeability and porosity measurements are not comparable with measurements of water penetration, they can give an indication of the latter as well. Air permeability and the quantity/shape of porosities provide indirect evidence of a concrete’s mechanical resistance during hydration and enable degradation phenomena to be assessed, especially for concrete and mortars in place. The well-known dependence of strength on porosity suggests that it can only be evaluated by means of Scanning Electron Microscopy (SEM) and mercury intrusion porosimetry (MIP) analyses on tiny specimens. In this study, we try to correlate the air permeability and the size of pores developing during the hydration of fast-setting mortars with the continuous growth of CSH crystals [20]. We collected the results of a gas permeability measurement campaign on cement mortar specimens, obtaining MIP measurements and SEM observations on the same samples at the same curing time.

Superabsorbent polyacrylates (SA-PA) were used as internal curing additives [14,21]. We investigated the effects of adding SA-PA, both to the fresh concrete and in the early stages of curing. Adding SA-PA in the amount of 0.5% wt of a dry powder mix seemed to modify the concrete’s setting and hardening. Our systematic experimental study on its porosity generated some interesting conclusions regarding the differences between the cement paste’s permeability in air and water.

## 2. Materials and Methods

The pre-mixed powder containing cement and quartz CONCENTRATE XYPEX, provided by PROBAR Italia (Cormano, Milan, Italy) had a phase composition as in Table 1. A mortar was prepared to add 1 part of H_2_O to 5 parts of pre-mixed powder, starting to release heat. The mix was poured immediately into petri dishes, which were covered and vibrated for 30 s. The hardened specimens were disks 15 mm thick with a 75 mm average diameter. They were cured for different times in a conditioned environment at (20 ± 2) °C and R.H. (50 ± 5)%. Each specimen was tested immediately after its curing was over.

A single batch of commercial superabsorbent sodium polyacrylate (SA-PA) was used for all the experiments in the amount of 0.5% wt. of dry powder and accurately mixed before adding water and then stirring for a few seconds before casting. The polymer was not brought to the dry state because it is hygroscopic, and its handling would have made it impossible to exactly quantify the adsorption of moisture from the air.

The particle size analysis was done in anhydrous acetone with a Delsa Nano C Particle analyzer from Perkin Elmer™ (Milan, Italy). The particle size distribution of the SA-PA was around 0.1–10 µm in diameter while the Xypex mortar particles varied from 0.5 to 0.9 µm.

Spreading tests were conducted by pouring the two mixtures, with and without SA-PA, in a cylinder 21 mm in diameter, adding a fixed volume of ~7.4 mL of the mixtures. The cylinder was then lifted rapidly to allow the specimen to settle. The procedure was performed on a PET transparent sheet so that the diameter could be measured after the mortar had set. Three different tests were run for each sample, and four different diameters were measured with a caliber for each test.

The permeability coefficient of the mortar specimens without SA-PA was measured according to UNI 1116400:2005, using compressed air with a dynamic viscosity = 1.8 × 10^−5^ Pa·s, applying a pressure gradient and detecting its flow rate and inlet pressure. The permeability characteristics were ascertained assuming the stationary flow conditions needed to apply the Hagen–Poiseuille relationship, which governs the permeation of compressible fluids in a microporous body. The sample was initially placed in the measuring cell at atmospheric pressure. Then, the gas was delivered to the cell at different pressures, and the gas flow rate at each pressure value was measured every 5 min until a difference of less than 3% was reached, then a final reading was taken. K was calculated according to the Hagen–Poiseuille equation: K = 2∙Q∙p_0_∙H∙η/[A∙(p^2^ − p_a_^2^)] [m^2^], where Q is the flow rate, p_0_ the pressure at which the measurement is carried out, H the specimen thickness, η the dynamic viscosity, A the specimen’s cross section orthogonal to the flow, and p and p_a_ the gas pressures at the inlet and outlet, respectively. Permeability coefficients were calculated for different values of the pressure gradient in the range of 20–100 kPa and the average permeability coefficient of a specimen was the mean of the resulting values.

Fragments taken from the specimens at different times were used for SEM (Coxem, Daejeon, Korea) observation. They were placed on stubs after lapping (some fragments were also observed before lapping) and coating with sputtered gold [22,23]. The COXEM EM30AX, supplied by Media System Lab, was used for the SEM observations. 

Quantitative phase analyses on the cement were carried out using X-ray diffraction (XRD). The patterns were collected using Thermo X’TRA diffractometer (Thermo Electron Corporation, Ecublens, Switzerland) in Bragg–Brentano geometry, with Mo-Kα radiation operated at 45 kV and 40 mA. XRD spectra were analyzed by TOPAS 7 software (Coelho Software, Brisbane, Australia) based on the crystalline database of American Mineralogist Crystal Structure.

Porosity and apparent density were measured with a mercury intrusion porosimeter, with increasing pressures on the fragments until they filled with mercury [24]. Applying an external pressure, and given its non-wetting property, mercury penetrates through the open porosities of a solid sample. The quantity of mercury that has penetrated the pores at each pressure step is measured, and the cumulative volume is obtained as a function of the pore radius [25,26]. The standard deviations of all the data are less than 0.05 and only the average value of each distribution was shown in the final graph.

Dynamical mechanical spectroscopy (DMS) tests were carried out on the different formulations during the first days of curing using an EXSTAR DMS 6100 (RT Instruments, Woodland, CA, USA) with the shear stress module, applying four load–unload cycles of 5 N with a load rate of 500 mN/min and measuring the deformation. Each specimen had a fixed dimension of 1 cm^2^ and a thickness of ~3 mm for a total shear plane of 30 mm^2^.

All the experiments have been carried out at least 3 times to ensure reproducible results. Only MIP were carried out twice due to a long test that might cause the sample to change especially during the first days when the hydration process is fast.

## 3. Results and Discussion

### 3.1. Mortar with a Crystallization Admixture 

The mortar was prepared using a commercial fast-setting blend, the composition of which (based on X-ray diffraction) is shown in Table 1. It contains mainly quartz sand, C_3_S (hartrurite), C_2_S, C_4_AF (brownmillerite) and CaSi_2_O_5_ (CS_2_ is the abbreviation used in cement chemistry) to quickly activate hydration reactions, produced at a lower temperature and higher pressure than those of Portland cement phases [27,28]. A lime-silica mixture, obtained by firing at around 900 °C and then quenching, reacts hydraulically faster than Portland cement, via a pozzolanic reaction in pozzolan/lime mixtures.

**Figure 1:**
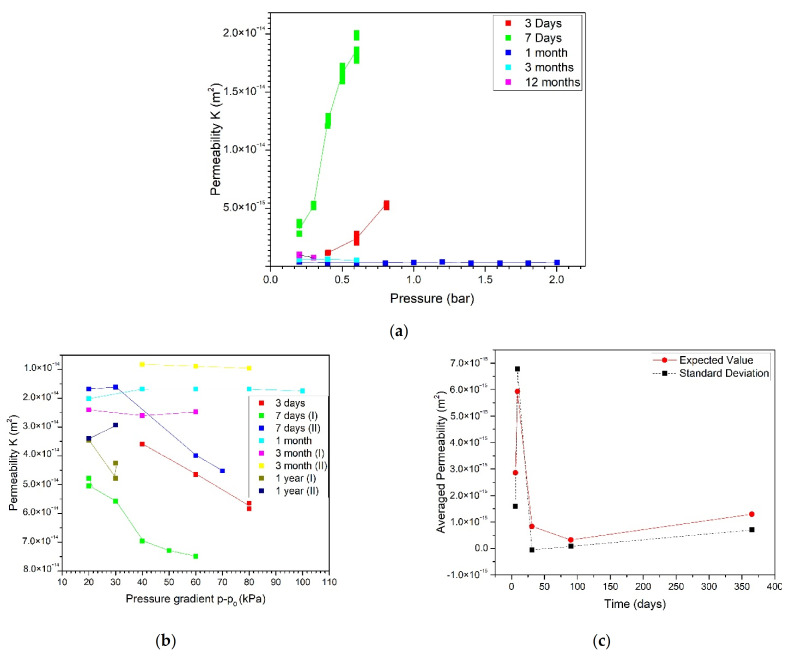
Permeability (K) at different curing times vs. pressure (**a**) and pressure gradient (**b**); average permeability vs. time (**c**).

Figure 1 shows the trend of the permeability (K) with the pressures and curing times. Figure 1a shows the increase in K about a week after mixing, followed by a marked reduction within a month. Figure 1b shows even more clearly that K is highly sensitive to the pressure gradient for up to 7 days of curing. In Figure 1c, the coefficient of average permeability is shown over different hardening times. Its value was estimated as the average value over different pressures according to UNI 1116400:2005. The average permeability was 2.96 × 10^−15^ m^2^ after 3 days, peaked at 6.04 × 10^−15^ m^2^ after 7 days, and then dropped to 3.07 × 10^−17^ m^2^ after about one month. Then, it varied slightly over time (4.13 × 10^−16^ m^2^ after 3 months, and 1.37 × 10^−15^ m^2^ after 1 year), and so did its standard deviation over different pressures (Figure 1c). In fact, the average permeability had higher values after about a week than after longer curing times, varying between 4% and 8% for the same specimen at the same point on the pressure gradient. After about a month, time no longer affected permeability, it became independent of the pressure gradient and its variability, for the same sample at the same pressure gradient, dropped well below 4%. These results can be explained by the mechanism of cement hydration. The starting mixture consists of cement powder and water. Three or seven days after mixing, hydration is still poor and, when the specimen undergoes air permeability testing, the remaining free water goes away, leading to a high porosity. Over time, crystallization proceeds, consuming water and reducing the mixture’s pore size [29].

**Figure 2:**
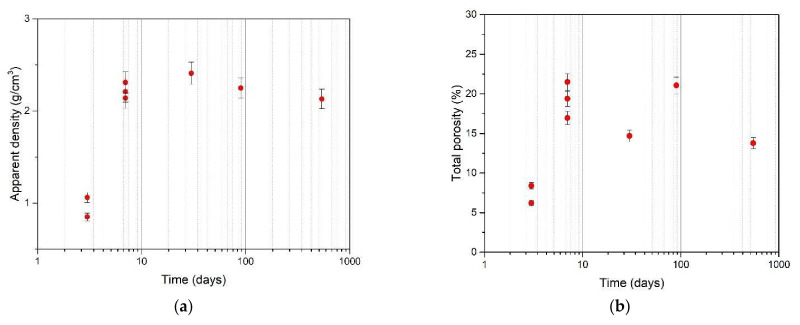
Apparent density (**a**) and porosity percentage (**b**) at different times (The instrumental error (2%) is applied to the data).

The apparent density and porosity follow the trends shown in Figure 2, as the hydration/crystallization process underway modifies the nature and microstructure of the mixture as it passes from the fresh to the hardened phase [30]. This change in the cement phases due to crystallization appears more clearly in SEM micrographs of mortars at different times. The samples’ appearance changes at both low and high magnification.

**Figure 3:**
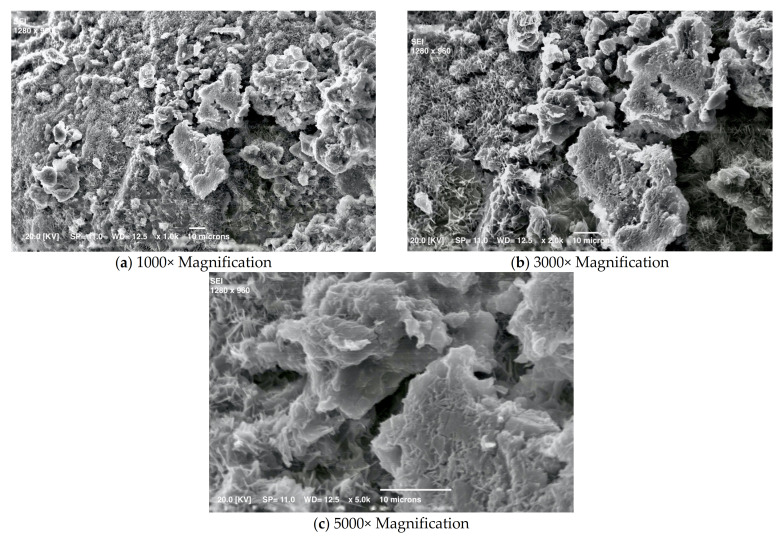
SEM micrographs of mortar at one day.

**Figure 4:**
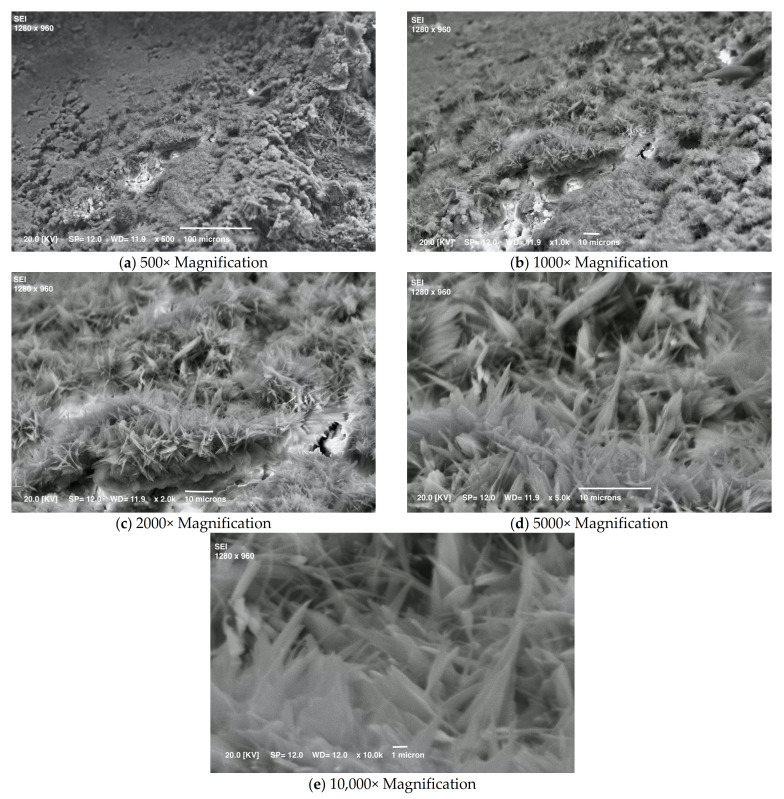
SEM micrographs of mortar at three days.

**Figure 5:**
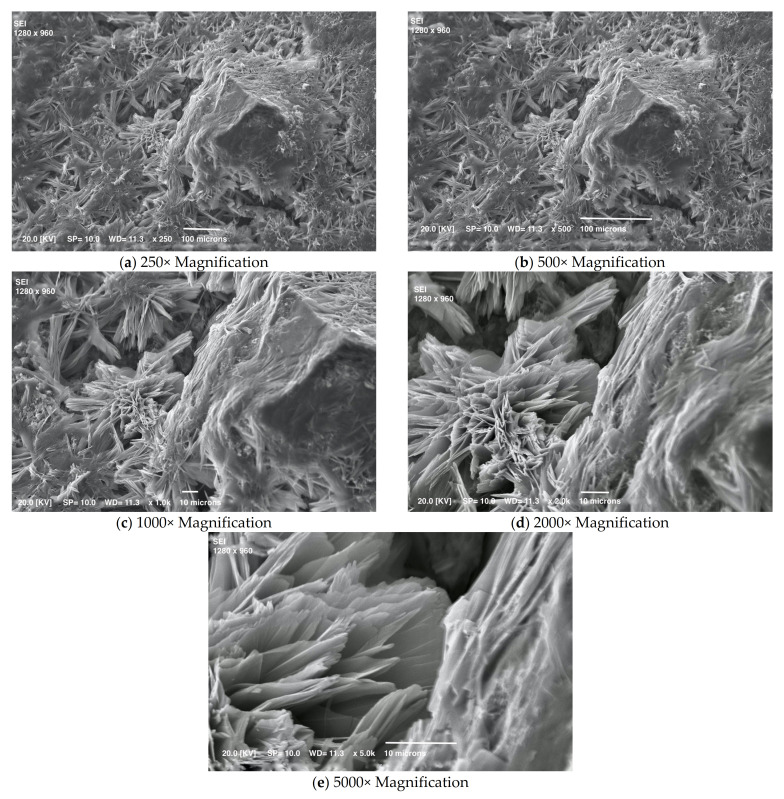
SEM micrographs of one mortar at one month.

**Figure 6:**
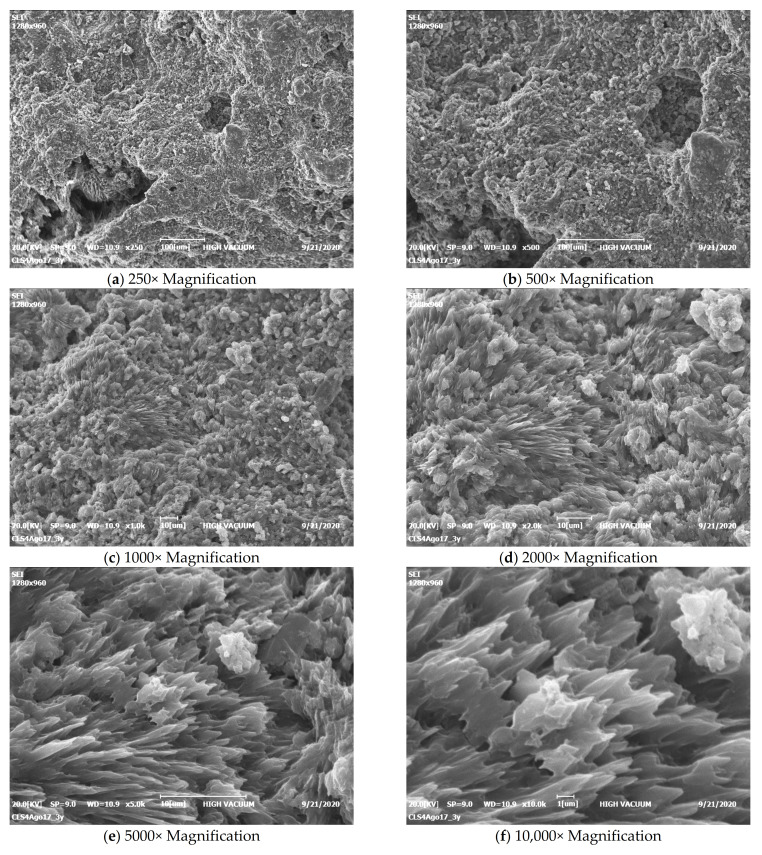

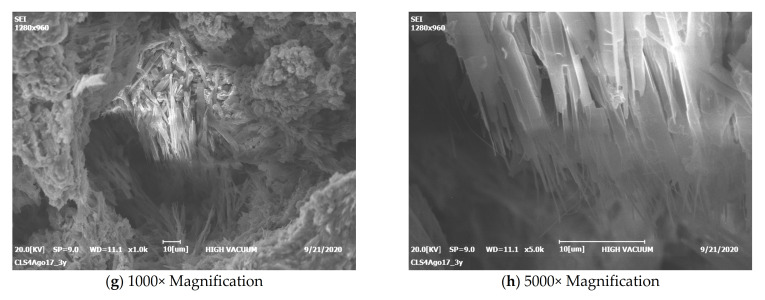
SEM micrographs of mortar after 12 months (non-polished sample).

**Figure 7:**
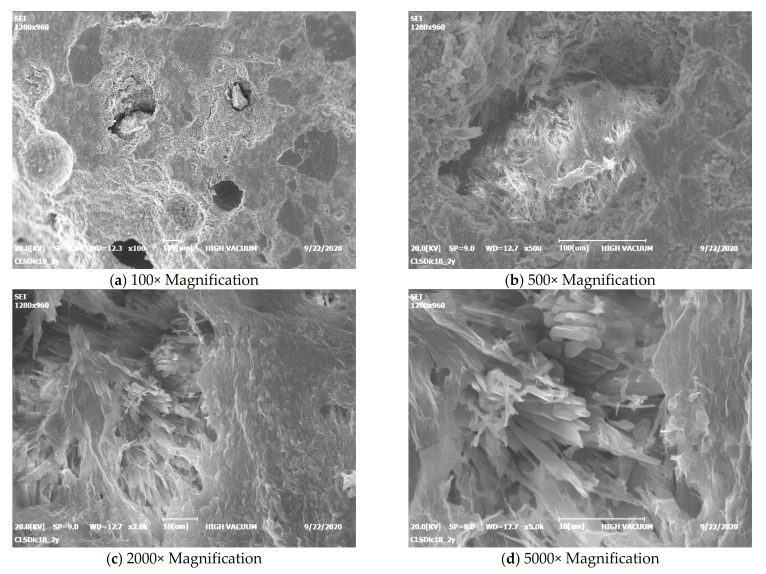

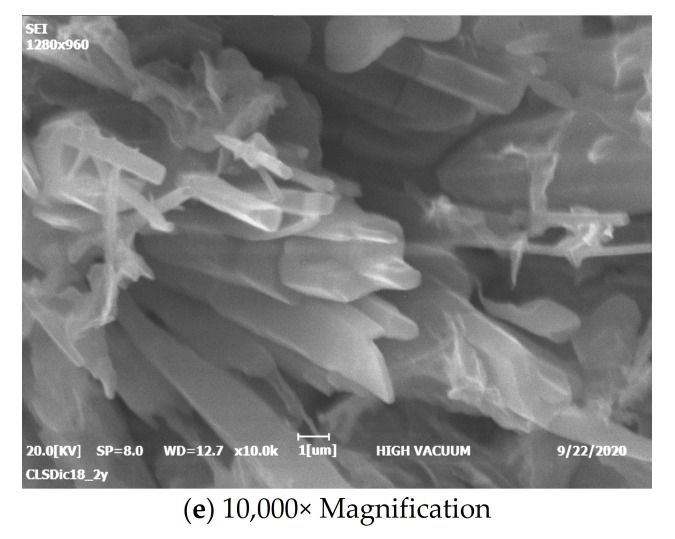
SEM micrographs of mortar after 18 months (pore).

Figure 3, Figure 4, Figure 5, Figure 6 and Figure 7 show details of the changes that occur. Small crystals are already visible after one day of curing, but they change in shape and size with time. The mortar’s early crystallization is facilitated by the fast-hydrating phases, which act as crystallization seeds and catalyze the nucleation of C-S-H in the capillary pores of the hydrating cement paste [31]. Moreover, Figure 4 shows numerous very small crystals on the surface, which by this time could also be calcium carbonate formed due to the reaction of portlandite in the presence of CO_2_.

It is worth noting how different the sample looks from all the others after 1 month, using the same magnifications (Figure 5). Lamellar crystals can be seen, which by this time must be CSH, still free to grow outwards in free space with no major constraints [32]. Figure 6 shows that, after a year, the hardening phase is fairly complete, as the surface is already evenly covered with lamellar crystals forming a compact and interconnected whole; the crystals can no longer grow freely, but only inwards where there is a residual empty space. Looking inside a pore (the last two images of Figure 6), we can see a group of crystals elongated in the only direction still without constraints. These features were visible because the sample was not polished before SEM observation. 

After 18 months, the crystals are so well developed and the empty spaces so greatly limited that crystals can only be seen on the fracture surfaces and inside the macropores (Figure 7).

In all the samples, the cubic crystals typical of portlandite are missing. This could be mainly due to the pozzolanic reaction, S + H_2_O + CH → C-S-H, which takes place in a paste with a cement rich in silica [7], besides an early limited amount of surface carbonization. This ensures that the pozzolanic reaction can continue every time the mortar comes into contact with water or condensed moisture. New crystals thus accumulate inside capillary pores, becoming an integral part of the network, improving strength and durability [33,34].

The direct evidence of the crystals changing with longer hardening times is a purely qualitative measure of a mortar becoming stronger and less permeable. An indirect, but more quantitative, analysis can be conducted using MIP, which can provide information on total porosity, average pore size, etc. That said, pore size distribution, from macro- to meso- and microscopic, emerged as the most accurate (albeit indirect) way to assess the increase in impermeability. The size and number of macropores depend on the casting process and do not change much over time, whereas the distribution of micro- and mesopores depends directly on the crystallization process [35].

**Figure 8:**
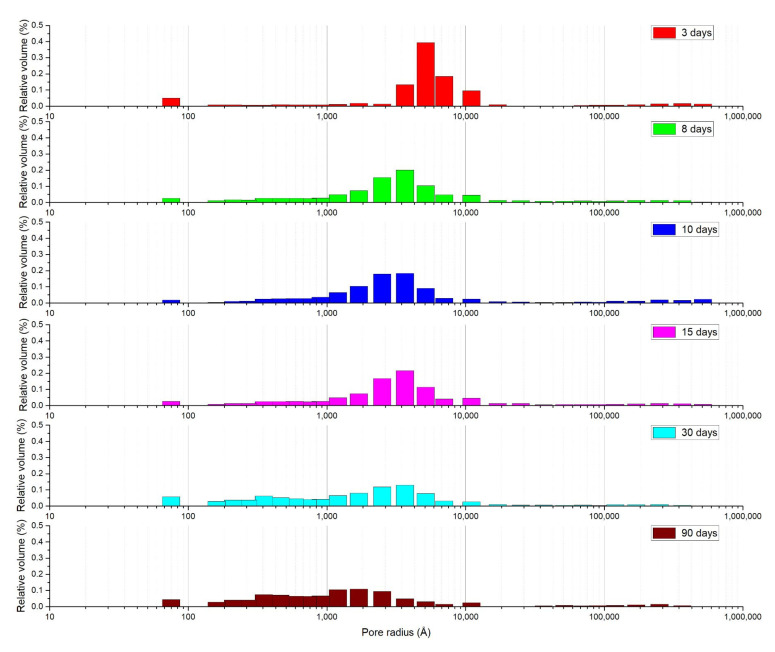
Pore size distribution in mortars at different times.

Figure 8 shows how the pore size distribution inside the concrete changes significantly over time, regardless of variability between samples and casting methods. The formation of inner hydration products among the previously formed outer ones reduces the proportion of large pores and increases that of meso- and micropores. The concrete’s porosity thus becomes increasingly discontinuous, thereby improving its impermeability. It is worth noting that the most significant changes in pore size distribution are apparent after about a week and then at one month. After a week, the larger pores seem to be reduced in number, giving rise to many mesopores, which improve interconnection and permeability. After a month, the micro (nano) pores increase with a consequent decline in pore interconnection and permeability.

This finding could also be of interest as an indirect measure of degradation [6]. As the deterioration phenomena occurring during a concrete’s service life depend on the average size of the pores, and the mesopores in particular, knowing the pore size distribution could be very useful in predicting the quality of a cement paste. MIP could therefore be used as a test to compare different concrete and examine the effects of different mix-designs. It could even be used to monitor the state of concrete structures during curing or in chemically aggressive environments, or at risk of physical deterioration due to freeze-thaw cycles, for instance. Combined with direct observation of the concrete’s surface using SEM, MIP could replace less easy permeability measurements on slices of concrete core extracted from part of the structure. SEM also lets us directly investigate the transition zone between the cement paste and aggregates, where porosity or microcracking would otherwise be undetectable.

Many approaches to reducing permeability are well known and should be used together, from proper mix design to prolonged curing, mineral additions and super-plasticizing or hydrophobic admixtures. The main factors for reducing permeability are the characteristics of the cement paste, the quality of the aggregate/paste interphase, and the paste/aggregate ratio, which must be low because aggregate is generally less porous than cement paste [36].

### 3.2. Mortar with a Crystallization Admixture: Effect of SA-PA Modification

A superabsorbent polyacrylate is a type of polymer with a strong water absorbing and water retaining capacity [37,38,39]. Strongly hydrophilic groups, such as carboxyl (–COOH) or hydroxyl(–OH) on the main chain and the graft side chain enable the polymer to absorb several thousand times its own weight, forming a gel with very peculiar features. Adding SA-PA to cement could supply it with moisture for internal curing. This common use was first considered due to the relatively large amount of energy required by the evaporation of adsorbed water, which reduces the loss of free water from fresh concrete. However, being highly hygroscopic, SA-PA removes water from the cement during mixing, reducing the fluidity of the fresh mix. The “slump flow” test setup for self-levelling concrete (modified and adapted for the present study on this quasi-liquid mortar as described in the Experimental Section) was used to measure the diameter of the paste contained in a 7.4 mL volume cylinder poured onto a plate. The average size of the disks of plain mortar was 52 mm ± 1, much larger than the 39 mm ± 1 of the mix containing SA-PA. The latter acts as a viscosity modifying agent (VMA), which helps to prevent segregation, but could slowly release water over time and interact with the ionic species released, such as Ca^2+^. After 24 h, the specimen without SA-PA was already hard, while the one containing SA-PA could still be scored with an indenter. This delayed setting confirms the fast absorption of water by SA-PA, which prevents the crystallization additive from hydrating and forming seeds [31].

**Figure 9:**
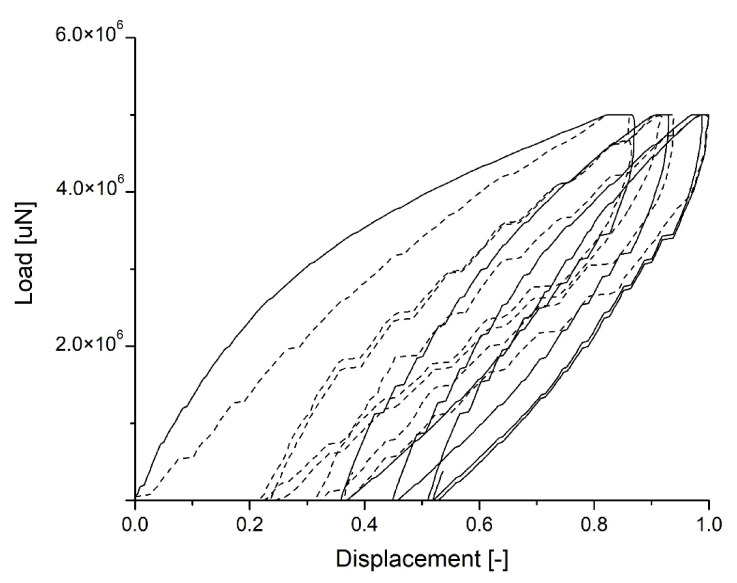
Cyclic load vs. displacement in mortars after seven days of curing: standard mortar (continuous line), mortar with SA-PA (dotted line).

In Figure 9, the load vs. displacement curves show an elastic-viscous-plastic behavior, demonstrated beyond the initial section by the presence of a hysteretic cycle, which suggests inelastic phenomena. It is worth noting that, along with loading and unloading at each cycle, there is approximately the same average slope, and some residual deformation accumulates with each cycle (though it seems to decrease with more cycles) and the hysteretic cycle seems to stabilize. These curves illustrate the difference between the two specimens, and the plain mortar’s less viscous behavior at 7 days compared with the sample containing SA-PA.

**Figure 10:**
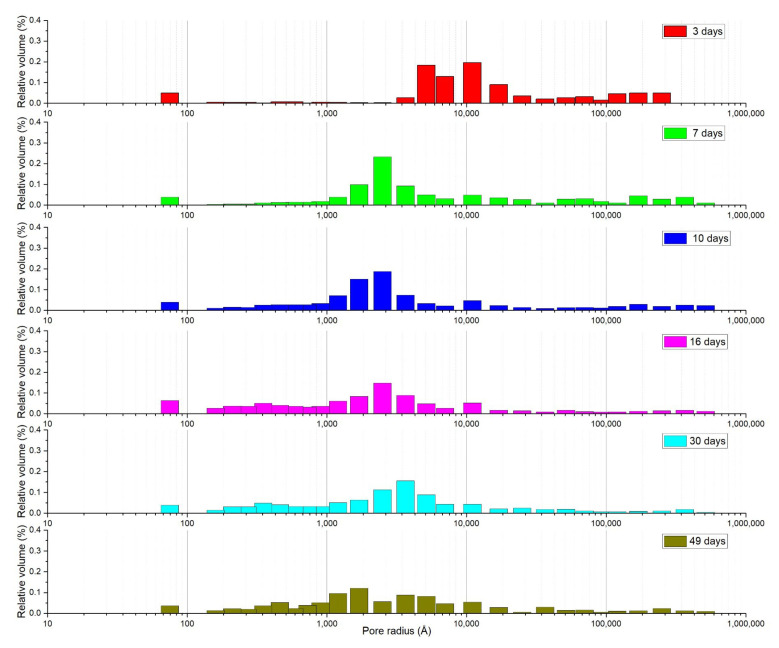
Pore size distribution in SA-PA modified mortars at different times.

The microstructural changes occurring in the cement paste during curing should therefore differ in the presence or absence of SA-PA. It has been reported [14] that water can be released by a difference in pressure or humidity between the internal capillaries of the hardened cement paste and the internal curing material. Due to the self-drying phenomenon, there is a pressure difference between the concrete pores and the pores of the internal curing material, so the water flows from the latter to the hardened paste [14]. According to Fick’s law, the moisture in the internal curing material spreads to the hardened cement paste due to the difference in humidity, and the flow rate of this diffusion is proportional to the relative humidity gradient [14]: the greater the relative humidity gradient between the two, the greater the diffusion. This makes the effects of internal curing materials more significant. In fact, the concrete’s internal porosity reportedly seemed to be greatly improved after adding SA-PA, without any actual increase in its permeability [14]. Accordingly, the evolution in the pore size distribution for mortar containing SA-PA, shown in Figure 10, clearly reveals a big difference compared with plain mortar: after 3 days of curing, the pores are larger. By 7 days, the pore size distribution has changed considerably, but then remains almost identical for about a month, with a slight increase in the proportion of micropores over a longer time. This important result suggests that, to estimate a concrete’s water permeability, it is not enough to simply measure its permeability in air. In fact, the air permeability of the mortar containing SA-PA remained greater than that of the plain mortar, whereas its water permeability even increased due to absorption and closing of the pores [14]. A greater air permeability, along with the evaluation of pore size distribution, could help to reduce spalling in high-temperature concretes [40,41]. The evolution of the pore size distribution charted in our study also shows that SEM observations need to be conducted at very high magnification levels in order to grasp crystallization over time, otherwise the technique is unable to reveal changes taking place at the micro (nano) level.

## 4. Conclusions

A mortar was prepared using a pre-mixed powder containing cement, quartz, portlandite and calcium silicates, combined with water and cast in petri dishes to obtain a number of samples for different analyses at increasing curing times. The increasing crystallization was monitored visually using SEM, which showed how lamellar crystals initially grew outwards and became longer, then changed in shape and grew inwards with longer curing times, when there was no more space available. The reduction in the mortar’s permeability was tested under the hydrostatic pressure of air, according to Hagen-Poiseuille. The mortar’s permeability increased almost linearly with the pressure gradient in the first week after mixing. It reached its maximum average value after about one week (6 × 10^−15^ m^2^), when some unreacted cement and free water were still present, coinciding with a high porosity during the test. Then, it decreased significantly to 1 × 10^−15^ m^2^. The growth of C-S-H crystals refined the capillary porosity and pore size distribution of the cement paste, as demonstrated by MIP measurements. The distribution of micro- and mesopores inside the mortar changed significantly, and the proportion of larger pores decreased with longer curing times. This would make the material more resistant to chemically aggressive environments or freeze–thaw cycles. Concrete deterioration depends on the average size of the pores, and the mesopores in particular, so testing pore size distribution could be used as a non-destructive test for assessing a concrete’s strength and its chemical and physical resistance. Early crystallization of the mortar was facilitated by the admixture in the pre-mixed powder, which hydrates quickly, forming crystalline seeds that act as nucleation sites [31].

This picture changes significantly when SA-PA is added as an internal curing material in the amount of 0.5% of dry powder. SA-PA also acts as a VMA, so its first observable effect is to reduce the slump flow of the fresh mix. As it sets, the fresh mix continues to behave differently from a mix without SA-PA in that it retains a residual plasticity after one day. The main difference, however, could be seen in the evolution of the pore size distribution. After 3 days, large pores were prevalent in the hardened mix, but this distribution shifted towards a prevalence of mesopores within a week, then remained virtually unchanged for about a month, with a slight growth of small pores due to crystallization. In the light of our findings, and the literature, this would suggest an effect of SA-PA in blocking the penetration of water, although permeability to air remains, and could even be useful for high-temperature concrete.

## Figures and Tables

**Table 1:** Phase composition of Xypex mortar.

Phase	%	Error
Quartz	11	1
Portlandite	13	1
CS_2_	14	1
Hartrurite	26	1
C_3_A	2.8	0.1
C_2_S	11	1
Gypsum	2	1
Brucite	5	0.3
Calcite	5	1
Kyanite	3	1
Arcanite	2	0.3
Brownmillerite	3	0.3
Anhydrite	2	1
Crystalline Index *	4	

* Ratio between the amorphous and crystalline area.

## Data Availability

Not applicable.
